# The association between vitamin D intake and the prevalence and mortality of asthma in the US adults

**DOI:** 10.1186/s12937-025-01171-z

**Published:** 2025-07-02

**Authors:** Yu Lei, Yu Luo, Yi Wang, Caiyang Liu, Lei Luo, Ji Li

**Affiliations:** 1 Department of Cardiothoracic Surgery, The First People’s Hospital of Neijiang, Sichuan, 641000 China; 2 Department of Geriatrics, The First People’s Hospital of Neijiang, Sichuan, 641000 China

**Keywords:** Vitamin D intake, Asthma, Prevalence, Mortality, NHANES

## Abstract

**Background:**

The relationship between vitamin D intake and asthma prevalence, as well as the control of asthma symptoms, remains a contentious issue in clinical settings. This study aims to clarify the relationship between vitamin D intake and asthma outcomes.

**Methods:**

In this study, three logistic regression models were employed to investigate the association between vitamin D intake and the prevalence of asthma. And a generalized additive model with a smoothing spline were utilized to explore the linear association. Additionally, subgroup analyses were conducted to assess potential effect modification by various factors. For asthmatic patients, the cox proportional hazards models were used to examine the relationship between vitamin D intake and mortality outcomes. And the restricted cubic splines were applied to assess non-linear relationships and threshold effect analyses were conducted to identify potential optimal levels of vitamin D intake that could significantly impact the risk of death from asthma. The data for this analysis were sourced from the National Health and Nutrition Examination Survey database.

**Results:**

The logistic regression analyses revealed a significant inverse association between vitamin D intake and asthma prevalence, with each unit increase in logarithmic vitamin D intake (Log VITD) associated with an 8% decrease in the risk of asthma (OR = 0.92, 95% CI: 0.86, 0.98, *p* = 0.02). Subgroup analyses showed a more pronounced inverse association in males (OR = 0.79, 95% CI: 0.71, 0.88, interaction *p*-value < 0.01). In the cohort of asthmatic patients, a non-linear relationship between vitamin D intake and all-cause mortality was identified, with inflection points at Log VITD values of 5.36 and 6.52. For respiratory disease mortality, an inflection point at Log VITD 5.48 was found, below which increasing vitamin D intake was associated with decreased respiratory disease mortality (HR = 0.01, 95% CI: 0.00, 0.05, *p* < 0.01), and above which it was associated with increased mortality (HR = 45.59, 95% CI: 17.61, 118.03, *p* < 0.01).

**Conclusion:**

This study demonstrates a inverse association between vitamin D intake and the prevalence of asthma, particularly in males. In the asthmatic population, there appears to be an optimal range of the Log VITD that may reduce mortality (ranging from 5.36 to 5.48). These findings suggest that maintaining appropriate levels of vitamin D intake may be beneficial for asthma management and potentially reduce asthma-related mortality.

**Supplementary Information:**

The online version contains supplementary material available at 10.1186/s12937-025-01171-z.

## Introduction

 Asthma, a prevalent chronic airway inflammation, impacts an estimated 262 million individuals globally, including a significant portion of US adults [ 1 ]. Marked by reversible obstruction and airflow limitations, asthma leads to considerable morbidity, frequent exacerbations, diminished quality of life, and high healthcare usage [ 2 ]. Its economic impact is substantial, with medical costs and productivity losses amounting to an annual burden of $81.9 billion in the United States [ 3 ].

The etiology of asthma is complex and multifactorial, involving a combination of genetic predisposition and environmental exposures. Known risk factors include allergen exposure, air pollution, tobacco smoke, and infections, among others [4]. Additionally, there is a growing body of evidence suggesting that adverse environmental and lifestyle factors, such as childhood adversity and psychological stress, may contribute to the development and severity of asthma [5, 6].

Vitamin D, traditionally recognized for its role in calcium homeostasis and bone health, has emerged as a potential modulator of immune function and inflammation [7]. The active form of vitamin D, 1,25-dihydroxyvitamin D (1,25(OH)2D), exerts immunomodulatory effects by influencing the expression of cytokines and antimicrobial peptides, potentially impacting the pathogenesis of asthma [8]. Vitamin D receptors are widely distributed, including in lung tissue, suggesting a direct role for vitamin D in pulmonary health [9].

The relationship between vitamin D status and asthma has been a subject of intense investigation. An observational study has reported lower vitamin D levels in asthmatic individuals, suggesting a potential protective role against asthma development or severity [10]. Conversely, another randomized controlled trial has found in children with chronic asthma and low vitamin D levels, vitamin D3 treatment did not significantly prolong the time to a severe asthma exacerbation when compared to a placebo [11]. Two meta-analyses have respectively found that vitamin D supplementation reduces the rate of asthma exacerbations requiring systemic corticosteroid treatment and that vitamin D supplementation can lower the incidence of acute asthma attacks in children [12, 13].

Given the inconsistent findings in the literature, the present study aims to investigate the association between vitamin D intake and the prevalence of asthma in the adult U.S. population using the National Health and Nutrition Examination Survey (NHANES) database. Furthermore, this study explores whether vitamin D intake is associated with mortality outcomes in adults with asthma, including all-cause mortality and mortality specifically attributed to respiratory diseases. It is hypothesized that higher vitamin D intake is associated with a lower prevalence of asthma in the adult U.S. population. Additionally, it is hypothesized that higher vitamin D intake is associated with lower all-cause mortality and lower respiratory disease-specific mortality in adults with asthma. Understanding these relationships could have important implications for asthma prevention and management strategies, particularly in the context of modifiable risk factors such as vitamin D intake.

## Materials and methods

### Data accession and study population

The data for this article were sourced from the NHANES, a program of the National Center for Health Statistics (NCHS) under the U.S. Centers for Disease Control and Prevention. NHANES conducts ongoing surveys combining interviews and physical examinations to assess the health and nutritional status of adults and children in the United States, examining a nationally representative sample of about 5,000 individuals annually, with data released in two-year cycles. The survey collects demographic, socioeconomic, dietary, and health-related information, along with medical and laboratory tests conducted by trained personnel. Research protocols were reviewed and approved by the NCHS Research Ethics Review Board, ensuring compliance with ethical standards. Participants were informed about the study’s objectives and risks, providing voluntary and informed consent to protect their rights and well-being.

**Fig. 1:**
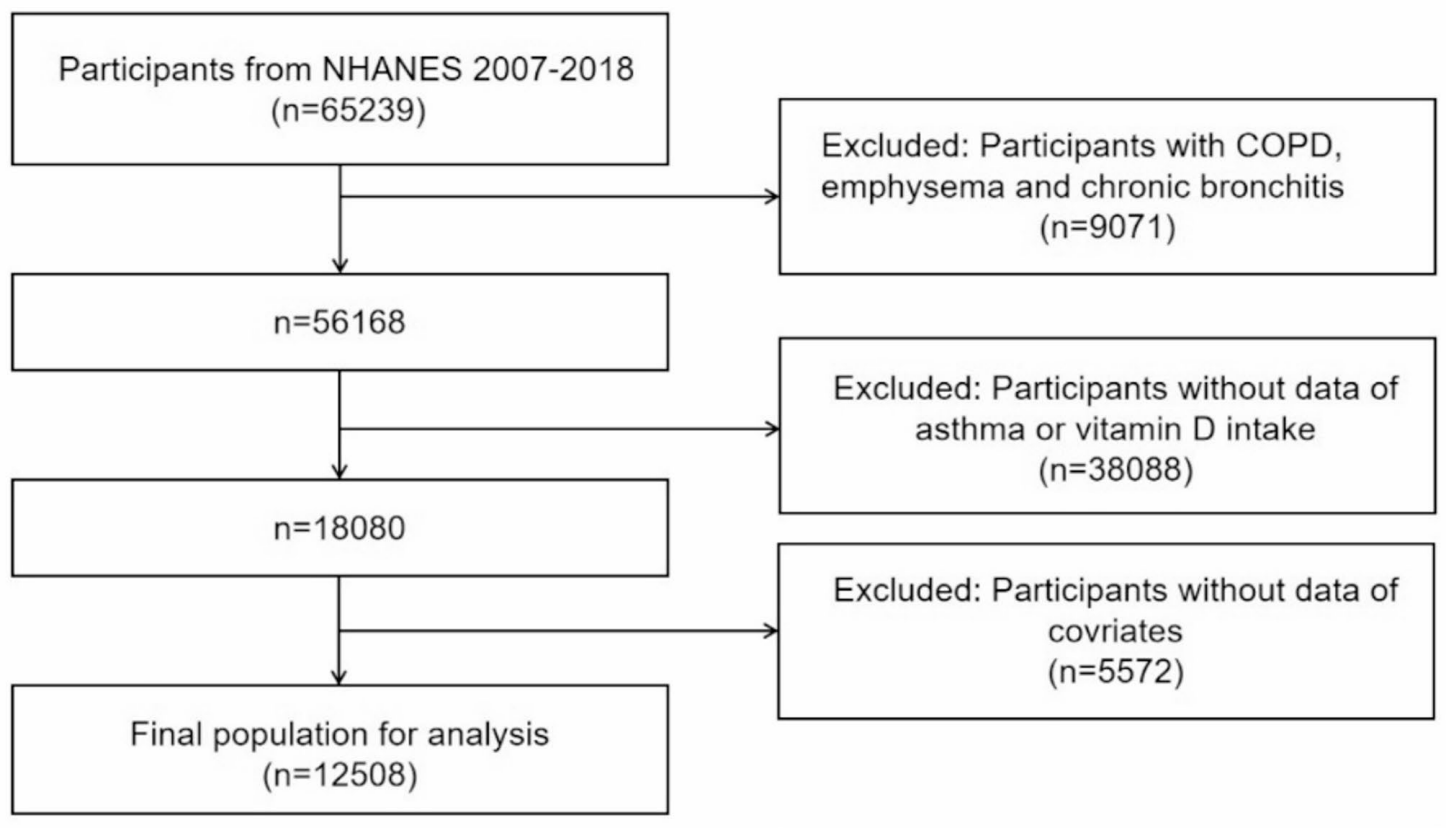
Participant inclusion flowchart for this study

Data from six cyclical years spanning from 2007 to 2018 were utilized in this study, encompassing a total of 65,239 adult participants (aged 20 and above). Participants with chronic obstructive pulmonary disease (COPD), emphysema, or chronic bronchitis were excluded (*n* = 9,071). Additionally, individuals with missing data on asthma and vitamin D intake were excluded (*n* = 38,088), as were those with incomplete covariate data (*n* = 5,572). Ultimately, 12,508 eligible participants were included in the analysis, with the screening process outlined in Fig. 1. Among them, there were 10,603 individuals without asthma and 1,905 asthmatic patients. Survival analysis was conducted using these asthmatic patients.

### Vitamin D intake calculation

The method for calculating the average daily vitamin D intake from the NHANES involved averaging the vitamin D intake from two consecutive 24-hour dietary recalls and adjusting for dietary supplements taken over a 30-day period. Specifically, the calculation was performed by adding the vitamin D intake from the 24-hour dietary recall on the first day to that of the second day, dividing the sum by 2, and then adding the average daily intake from dietary supplements calculated by dividing the total intake over 30 days by 30. This approach provided a comprehensive estimate of an individual’s daily vitamin D intake from both food and supplements.

Let V24h1 be the vitamin D intake from the first 24-hour dietary recall, and V24h2 be the vitamin D intake from the second 24-hour dietary recall. Let VSupp be the total vitamin D intake from dietary supplements over a 30-day period.

The average daily vitamin D intake VITD could be calculated as:

$${\rm VITD}\, ({\rm microgram},\, {\rm mcg}) = ({\rm V24h1}+{\rm V24h2})/2 + {\rm VSupp/30}$$

Regarding the two primary methods used in NHANES to measure vitamin D intake:

1. 24-Hour Dietary Recalls: This method captured the types and amounts of foods and beverages consumed during the 24 h prior to the interview. Vitamin D intake was estimated from the foods reported by survey participants, which included the sum of ergocalciferol (Vitamin D2) and cholecalciferol (Vitamin D3) content. Since 2007, the 24-hour dietary supplement data were collected during the 24-hour recalls, allowing for the combination of nutrients from all sources.
2. Dietary Supplement Questionnaire: This involved collecting information on all vitamins, minerals, herbals, and other dietary supplements consumed during a 24-hour period, including the name and the amount of dietary supplement taken. The data from the 30-day dietary supplement intake files, which had different reference periods and measurement error characteristics, required some data manipulation to be combined with 24-hour recalls. The actual amount of nutrient consumed from supplements was calculated by dividing the participant’s reported amount taken by the serving size from the product label and then multiplying by the ingredient amount for each dietary supplement.

### Assessment of asthma and mortality

The diagnosis of asthma was based on two primary criteria: The first criterion was from the questionnaire data, where participants were asked if a doctor had ever told them that they had asthma (yes or no). The second criterion involved the pulmonary function test, specifically the bronchial dilation test. A positive bronchodilator response was defined as an increase in the forced expiratory volume in one second (FEV1) by 12% or more from the baseline value after the administration of a bronchodilator, or an absolute increase in FEV1 of more than 200 milliliters (ml). Participants who met either of these criteria were diagnosed with asthma.

The mortality data for this study was derived from the National Death Index (NDI), which was a systematic compilation of mortality records maintained by the NCHS and was used to ascertain the vital status of NHANES participants. This research utilized the 2019 Public Access Link mortality dataset, where personal identification information of NHANES participants aged 18 and above, including name, gender, date of birth, etc., was matched between the NHANES and NDI databases through a probabilistic matching technique. The calculation of mortality data involved identifying deceased survey participants and recording the cause of death from death certificates. The follow-up period was determined by taking the time from the date of the baseline interview and death or the end date of the study review, which was December 31, 2019, whichever came first.

### Covariates

The covariates included age (measured in years), gender (categorized as male or female), race (including Mexican American, other Hispanic, non-Hispanic Black, non-Hispanic White, and other races), education (less than 9th grade, 9-11th grade, high school graduate/GED or equivalent, some college or AA degree, college graduate or above), marital status (including married, widowed, divorced, separated, never married, living with partner), poverty income ratio (PIR), smoke (defined based on questionnaire responses, where individuals who have smoked more than 100 cigarettes in their lifetime and currently smoke are defined as smokers, and those who have not are defined as non-smokers), alcohol use [classified into non-drinkers (those who have not had more than 12 drinks in a year), moderate drinkers (12–48 drinks per year), and heavy drinkers (more than 48 drinks per year)], body mass index (BMI, kg/m^2), systemic inflammatory index (SII: a laboratory measure calculated as platelets multiplied by the ratio of neutrophils to lymphocytes), diabetes (self-reported based on questionnaire responses, categorized as yes, no or borderline), hypertension (self-reported based on questionnaire responses, categorized as yes or no), heart failure (self-reported based on questionnaire responses, categorized as yes or no), coronary heart disease (CHD, self-reported based on questionnaire responses, categorized as yes or no), albumin [ALB, measured in grams per deciliter (g/dL)], aspartate aminotransferase [AST, measured in units per liter (U/L)], alanine aminotransferase (ALT, measured in U/L), serum calcium [measured in milligrams per deciliter (mg/dL)], serum creatinine (Cr, measured in mg/dL), cholesterol (measured in mg/dL), triglycerides (measured in mg/dL), uric acid (measured in mg/dL). In the subgroup analysis, age groups were defined as under 40, between 40 and 60, and 60 and above, whereas BMI categories were set as under 25, between 25 and 30, and above 30 kg/m2.

### Statistical analysis

#### Study population description

The study population was characterized using descriptive statistics presented as mean ± standard deviation (SD) for continuous variables and N (%) for categorical variables. For continuous variables, the non-parametric Kruskal-Wallis rank sum test was employed to assess differences among groups due to the non-normal distribution of data. For categorical variables with theoretical counts less than 10, the Fisher’s exact test was utilized to determine statistical significance.

#### Log transformation of vitamin D intake

Given the non-normal distribution of VITD, a natural logarithm transformation was applied to the data, denoted as Log VITD, to normalize the distribution for further analysis. Specifically, the original VITD data exhibited a right-skewed distribution, with a long tail extending to the right. This skewness can violate the assumptions of parametric statistical tests, which typically require normally distributed data. By applying the natural logarithm transformation, the data were transformed to a more symmetric, approximately normal distribution. This transformation not only stabilizes the variance but also makes the data more suitable for linear regression analysis, which assumes a linear relationship between variables and normally distributed residuals. Therefore, the log transformation was deemed necessary to ensure the validity and robustness of the statistical analyses conducted in this study.

#### Exploratory data analysis

The relationship between Log VITD and asthma was initially explored using a generalized additive model (GAM) with a smoothing spline to confirm the linear association.

#### Regression analysis for asthma association

To validate the association between Log VITD and asthma, multiple logistic regression models were constructed. A crude model without covariates was initially assessed. Subsequently, an adjusted model I was developed, incorporating demographic covariates including gender, year, race, marital status, education, and PIR. A further adjusted model II was created by adding additional covariates such as smoking status, alcohol consumption, heart failure, CHD, diabetes, BMI, SII, ALB, ALT, AST, serum calcium, Cr, cholesterol, triglycerides, and uric acid. These models were used to confirm the relationship between Log VITD and asthma, and trend analyses were performed.

#### Quantile stratification and trend analysis

Log VITD was stratified into quartiles (Q1-Q4), and the association with asthma was assessed using the same set of regression models. Trend tests were conducted to evaluate the dose-response relationship across quartiles.

#### Subgroup analysis

Subgroup analyses were conducted to explore effect modification by gender, age, smoking, alcohol consumption, hypertension and diabetes. These analyses were adjusted using the covariates from the adjusted model II.

#### Cox proportional hazards models

Within the asthmatic population, the relationship between Log VITD and all-cause mortality, as well as respiratory disease mortality, was investigated using cox proportional hazards models. The same covariate adjustment strategy as in the logistic regression models was applied.

#### Restricted cubic splines (RCS) and threshold effect analysis

Non-linear relationships between Log VITD and mortality outcomes were assessed using RCS. Inflection points indicating potential threshold effects were identified, and separate analyses were conducted for different segments defined by these thresholds.

#### Multiple imputation for sensitivity analysis

To address missing covariate data, multiple imputation was performed, creating five imputed datasets (Imputation 1 to Imputation 5). These datasets were used to re-evaluate the non-linear relationship between Log VITD and respiratory disease mortality within the fully adjusted model II (including all covariates), and threshold effect analyses were repeated to ensure the robustness of the findings.

#### Weighted analysis

While NHANES survey weights are typically used to account for the complex survey design and ensure that the results are representative of the U.S. population, the application of these weights in certain advanced statistical models, such as the GAM and RCS, can be operationally challenging. Therefore, NHANES survey weights were applied only in the description of the baseline characteristics of the study participants to ensure representativeness. In the subsequent analyses, including the GAM, RCS, and Cox proportional hazards models, unweighted analyses were conducted to maintain the consistency and feasibility of the statistical methods. This approach acknowledges that the generalizability of the findings to the broader U.S. population may be limited, but it ensures the robustness and accuracy of the statistical techniques used.

All statistical analyses were executed utilizing Empower software version 4.2 and R version 4.2.1. Statistical significance was ascertained based on a *p* value criterion of below 0.05.

## Results

### Characteristics of participants

This study comprised a total of 12,508 participants, representing a weighted population of 591,281,998 individuals, including 1,905 individuals with asthma (representing 89,805,465 individuals) and 10,603 without asthma (representing 501,476,533 individuals). The average age of the participants was 59.03 ± 16.08 years, with 7,342 (58.70%) being female. Asthma patients tended to exhibit the following characteristics when compared to non-asthmatic participants: they were younger (55.18 ± 15.89 vs. 59.72 ± 16.02 years), had a higher proportion of females (65.62% vs. 57.46%), were more likely to be Non-Hispanic Black (22.68% vs. 17.69%), had a higher prevalence of individuals with some college or AA degree education (36.75% vs. 32.31%), were more often never married in terms of marital status (13.02% vs. 9.99%), had a lower PIR (2.80 ± 1.70 vs. 2.90 ± 1.62), exhibited a higher BMI (31.85 ± 8.39 vs. 29.66 ± 6.89 kg/m^2), were more likely to be smokers (48.45% vs. 45.45%), had lower levels of ALB (41.37 ± 3.49 vs. 41.91 ± 3.46 g/dL), higher levels of AST (25.66 ± 15.00 vs. 25.18 ± 11.90 U/L), lower levels of uric acid (321.97 ± 85.99 vs. 325.73 ± 85.82 mg/dL), and lower levels of VITD (566.62 ± 466.72 vs. 596.30 ± 479.82). Table 1 presents the baseline characteristics of the study population stratified by asthma status.

**Table 1:** Baseline characteristics of the study participants, weighted

Characteristics	Overall	Asthma		*p* value
		Without	With	
N(%)	12,508	10,603 (84.77)	1905 (15.23)	
Weighted population	591,281,998	501,476,533	89,805,465	
Age, years	59.03 ± 16.08	59.72 ± 16.02	55.18 ± 15.89	< 0.01
Gender				< 0.01
Male	5166(41.30)	4511(42.54)	655(34.38)	
Female	7342(58.70)	6092(57.46)	1250(65.62)	
Race				< 0.01
Mexican American	1326 (10.60)	1120 (10.56)	206 (10.81)	
Other Hispanic	935 (7.48)	786 (7.41)	149 (7.82)	
Non-Hispanic White	6561 (52.45)	5640 (53.19)	921 (48.35)	
Non-Hispanic Black	2308 (18.45)	1876 (17.69)	432 (22.68)	
Others	1378 (11.02)	1181 (11.14)	197 (10.34)	
Education				< 0.01
Less than 9th grade	766 (6.12)	639 (6.03)	127 (6.67)	
9-11th grade	1137 (9.09)	1000 (9.43)	137 (7.19)	
High school graduate/GED or equivalent	2677 (21.40)	2254 (21.26)	423 (22.20)	
Some college or AA degree	4126 (32.99)	3426 (32.31)	700 (36.75)	
College graduate or above	3802 (30.40)	3284 (30.97)	518 (27.19)	
Marital status				< 0.01
Married	7173 (57.35)	6106 (57.59)	1067 (56.01)	
Widowed	1537 (12.29)	1325 (12.50)	212 (11.13)	
Divorced	1573 (12.58)	1354 (12.77)	219 (11.50)	
Separated	347 (2.77)	273 (2.57)	74 (3.88)	
Never married	1307 (10.45)	1059 (9.99)	248 (13.02)	
Living with partner	571 (4.57)	486 (4.58)	85 (4.46)	
PIR	2.89 ± 1.63	2.90 ± 1.62	2.80 ± 1.70	< 0.01
BMI, kg/m^2^	30.00 ± 7.18	29.66 ± 6.89	31.85 ± 8.39	< 0.01
Smoke				0.02
Yes	5742 (45.91)	4819 (45.45)	923 (48.45)	
No	6766 (54.09)	5784 (54.55)	982 (51.55)	
Alcohol use				0.39
Never	4176 (33.39)	3543 (33.42)	633 (33.23)	
Moderate	8267 (66.09)	7001 (66.03)	1266 (66.46)	
Heavy	65 (0.52)	59 (0.56)	6 (0.31)	
Hypertension				0.39
Yes	5278 (42.20)	4457 (42.04)	821 (43.10)	
No	7230 (57.80)	6146 (57.96)	1084 (56.90)	
Diabetes				0.60
Yes	3334 (26.65)	2844 (26.82)	490 (25.72)	
No	8710 (69.64)	7368 (69.49)	1342 (70.45)	
Borderline	464 (3.71)	391 (3.69)	73 (3.83)	
Heart failure				0.80
Yes	667 (5.33)	563 (5.31)	104 (5.46)	
No	11,841 (94.67)	10,040 (94.69)	1801 (94.54)	
CHD				0.06
Yes	1085 (8.67)	941 (8.87)	144 (7.56)	
No	11,423 (91.33)	9662 (91.13)	1761 (92.44)	
SII	533.25 ± 350.00	532.11 ± 349.88	539.58 ± 350.68	0.57
ALB	41.83 ± 3.47	41.91 ± 3.46	41.37 ± 3.49	< 0.01
ALT	23.86 ± 14.79	23.75 ± 14.32	24.50 ± 17.15	0.19
AST	25.25 ± 12.42	25.18 ± 11.90	25.66 ± 15.00	< 0.01
Cr	84.00 ± 51.30	84.54 ± 54.56	81.00 ± 26.43	0.08
Cholesterol	4.84 ± 1.10	4.85 ± 1.11	4.82 ± 1.09	0.50
Triglycerides	1.75 ± 1.18	1.75 ± 1.17	1.75 ± 1.22	0.53
Uric acid	325.15 ± 85.85	325.73 ± 85.82	321.97 ± 85.99	0.01
Serum calcium	2.36 ± 0.10	2.36 ± 0.10	2.36 ± 0.10	0.38
VITD	591.78 ± 477.95	596.30 ± 479.82	566.62 ± 466.72	< 0.01

Continuous variables are expressed as the mean ± standard deviation, while categorical variables are presented as the number of cases (percentage)

### Association between log VITD and asthma prevalence

**Fig. 2:**
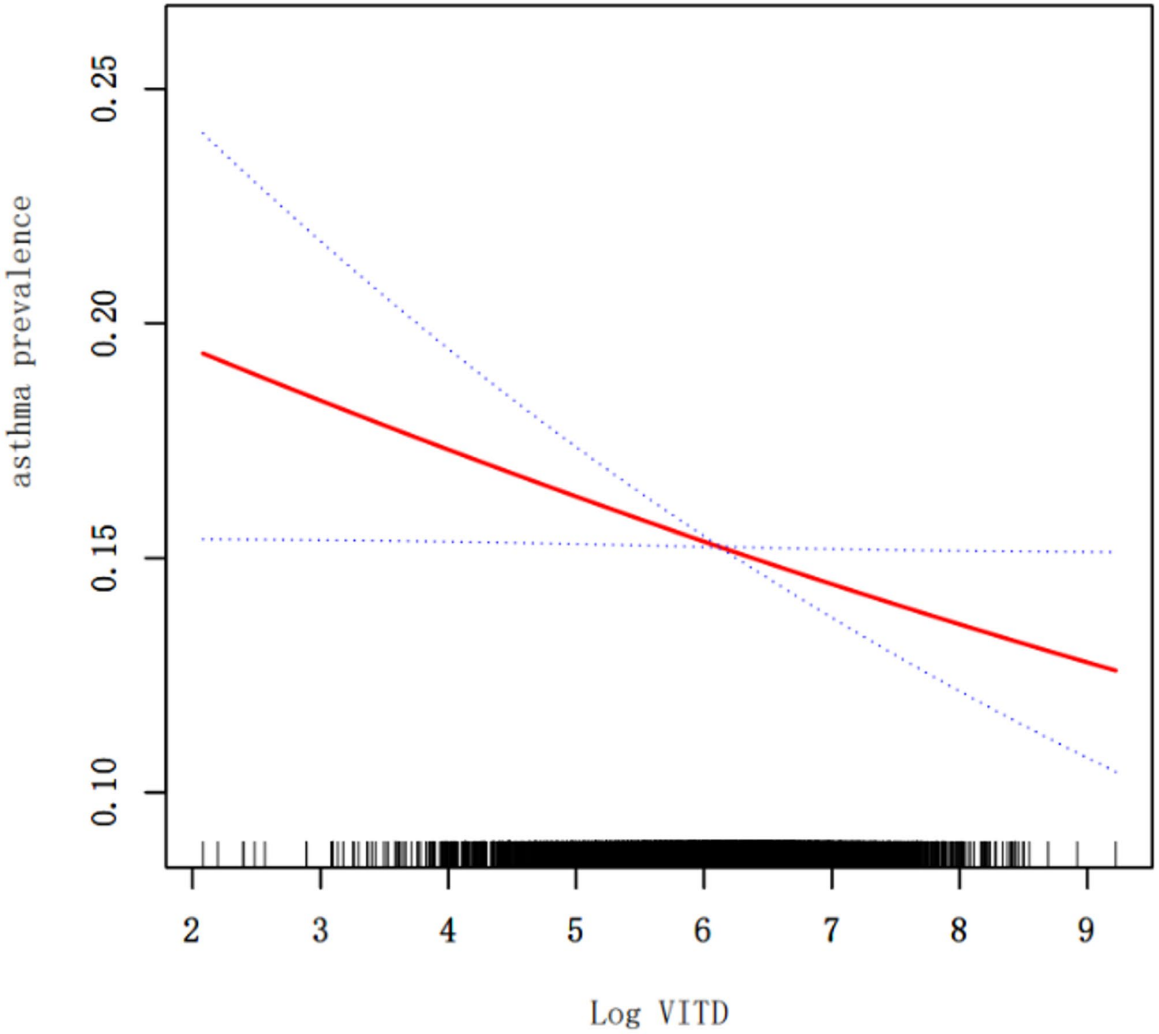
Linear relationship of Log VITD and asthma (GAM) The figure depicted the linear association between Log VITD and asthma prevalence, as modeled using a GAM with a smoothing spline. The solid red line represented the best-fit curve, indicating the estimated relationship between Log VITD and the log odds of asthma prevalence. The dashed lines represented the 95% CI, providing a measure of the precision of the estimated association. This analysis was conducted using the Adjusted Model II. Threshold effect analysis log-likelihood ratio: 0.16

The linear relationship between Log VITD and asthma prevalence was elucidated in Fig. 2, which was generated using a GAM with a fully adjusted model (Adjusted Model II). The GAM, supported by a log-likelihood ratio of 0.16 from the threshold effect analysis, confirmed the absence of inflection points, suggesting a linear association without any significant deviations. The curve indicated that higher values of Log VITD were associated with a lower risk of asthma prevalence.

Table 2 presented the results of the logistic regression analyses across three models. In the fully adjusted model, it was confirmed that for each unit increase in Log VITD, there was an 8% decrease in the risk of asthma prevalence (OR = 0.92, 95% CI: 0.86, 0.98, *p* = 0.02). Given the linear association between Log VITD and asthma, Log VITD was stratified into quartiles (Q1-Q4) and further analyzed. The highest Log VITD quartile (Q4) demonstrated a 22% lower risk of asthma prevalence compared to the lowest quartile (Q1) in the fully adjusted model (OR = 0.78, 95% CI: 0.68, 0.90, *p* < 0.01). The trend test for these quartiles was significant, with a *p*-value less than 0.05, reinforcing the dose-response relationship between Log VITD and reduced asthma prevalence. The ORs, 95% CIs, and *p*-values for all covariates in the adjusted models are provided in Table S1.

**Table 2:** Logistic regression analyses for log VITD and asthma

Exposure	Crude Model	Adjusted Model I	Adjusted Model II
	OR (95% CI) *p*	OR (95% CI) *p*	OR (95% CI) *p*
Log VITD (continuous)	0.90 (0.84, 0.96) < 0.01	0.96 (0.90, 1.03) 0.23	0.92 (0.86, 0.98) 0.02
Log VITD (quartiles)			
Q1	1	1	1
Q2	0.75 (0.66, 0.86) < 0.01	0.81 (0.70, 0.93) < 0.01	0.76 (0.66, 0.88) < 0.01
Q3	0.90 (0.79, 1.03) 0.11	0.99 (0.86, 1.13) 0.86	0.91 (0.80, 1.05) 0.20
Q4	0.72 (0.63, 0.83) < 0.01	0.82 (0.71, 0.94) < 0.01	0.78 (0.68, 0.90) < 0.01
*p* for trend	0.92 (0.88, 0.96) < 0.01	0.96 (0.92, 1.00) 0.08	0.94 (0.90, 0.99) 0.01

The crude model was not adjusted for any covariates, provided an initial estimate of the relationship. The adjusted Model I accounted for demographic covariates including age, gender, race, education, marital status, and PIR. Furthermore, Adjusted Model II included additional covariates such as smoking status, alcohol consumption, heart failure, coronary heart disease, diabetes, BMI, SII, ALB, ALT, AST, serum calcium, Cr, cholesterol, triglycerides, and uric acid

Q1-Q4 referred to the division of the Log VITD values into quartiles, each representing a 25th percentile range of the distribution

### Subgroup analysis

In the subgroup analysis, the fully adjusted model was utilized to assess the relationship between Log VITD and asthma prevalence across various subgroups, including gender, smoking status, alcohol use, hypertension, diabetes, age, and BMI. The analysis revealed that within the subgroups of males (OR = 0.79, 95% CI: 0.71, 0.88), individuals with moderate alcohol consumption (OR = 0.91, 95% CI: 0.84, 0.99), those with hypertension (OR = 0.86, 95% CI: 0.78, 0.95), participants without diabetes (OR = 0.88, 95% CI: 0.81, 0.95), individuals aged 60 years and above (OR = 0.85, 95% CI: 0.76, 0.94), and those with a BMI less than 25 (OR = 0.85, 95% CI: 0.74, 0.97), there were significant inverse associations between Log VITD and asthma prevalence.

**Fig. 3:**
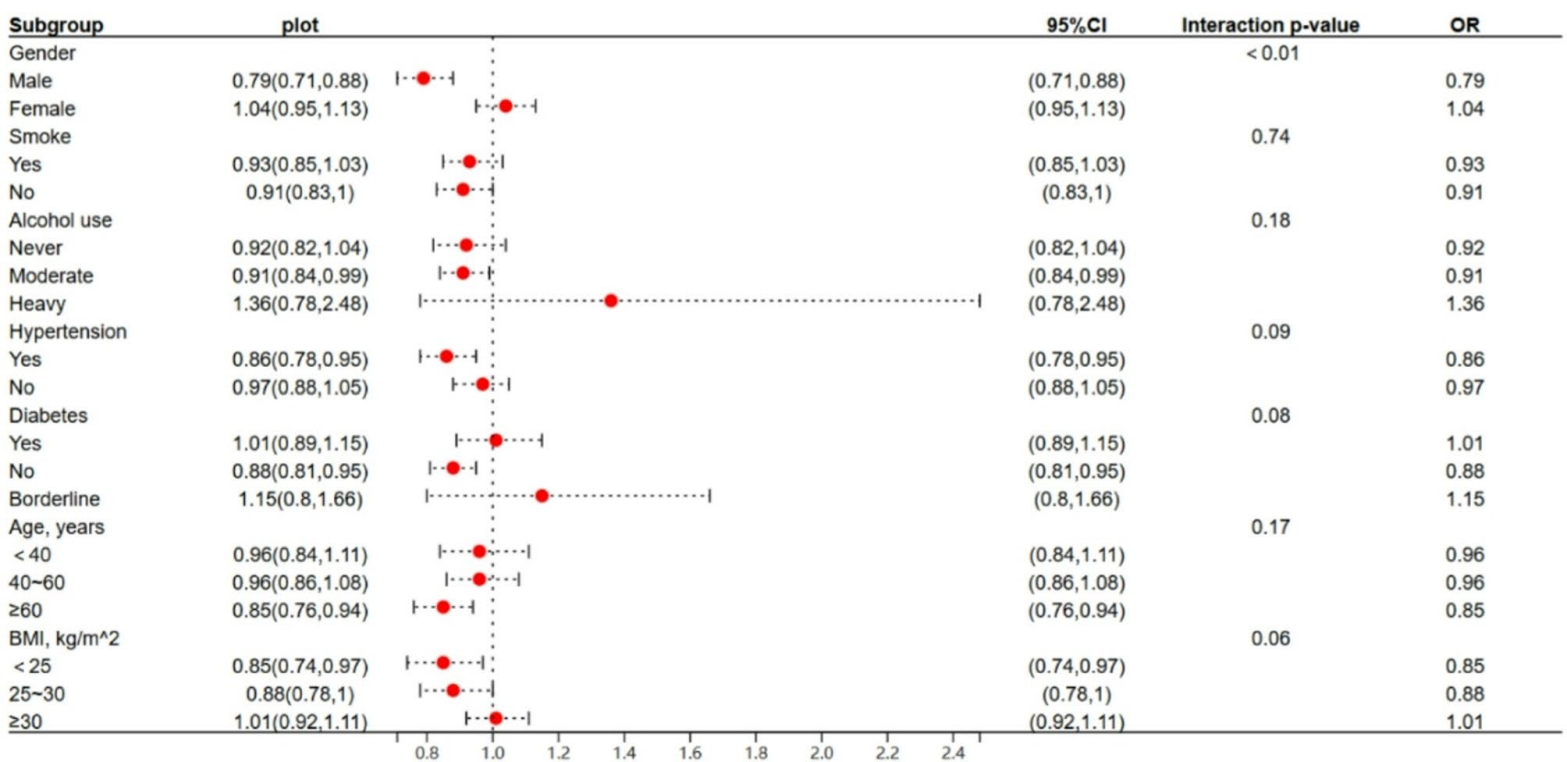
Subgroup analysis for the association between Log VITD and asthma

Subsequent interaction tests indicated that the difference in the association between Log VITD and asthma prevalence was statistically significant only between genders (interaction *p*-value < 0.01), while no significant differences were observed across the other subgroups. Specifically, within the male subgroup, a inverse association was observed between Log VITD and asthma prevalence. In contrast, within the female subgroup, no significant association was found between Log VITD and asthma prevalence (Fig. 3). Other factors, such as education level, race, and marital status, were not included in the subgroup analyses due to the limited sample size. Including these additional factors would have resulted in too many subgroups, each with a small sample size, which could compromise the statistical power and reliability of the analyses.

### Baseline characteristics of the asthma population

In the cohort study with a median follow-up time of 59 months (range 13–157 months), there were 1905 asthmatic patients, among whom 152 cases resulted in all-cause mortality. Compared to those who did not experience all-cause mortality, the deceased individuals were older on average (69.99 ± 9.97 years vs. 53.90 ± 15.66 years), had a higher proportion of females (53.29% vs. 46.71%), and a higher proportion of non-Hispanic individuals (84.21% vs. 69.88%). Additionally, a lower proportion of the deceased had a college degree or higher (47.37% vs. 65.37%). In terms of marital status, a significantly higher proportion of the deceased were widowed (25.66% vs. 9.87%). The prevalence of heart failure was markedly higher among the deceased (28.29% vs. 3.48%), as was the prevalence of CHD (16.45% vs. 6.79%). The deceased also had lower levels of ALB (39.41 ± 3.82 g/dL vs. 41.54 ± 3.41 g/dL), higher levels of AST (27.74 ± 10.60 U/L vs. 25.48 ± 15.31 U/L), and higher levels of Cr (97.96 ± 37.97 µmol/L vs. 79.53 ± 24.65 µmol/L). Lastly, although not statistically significant, the VITD levels were somewhat lower in the deceased group (559.56 ± 330.57 nmol/L) compared to those who did not die (567.23 ± 476.76 nmol/L, *p* = 0.06). For detailed comparisons, see Table 3.

**Table 3:** Baseline characteristics of the asthma population

Characteristics	Overall	All-cause mortality		*p* value
		No	Yes	
N(%)	1905	1753 (92.02)	152 (8.98)	
Age, years	55.18 ± 15.89	53.90 ± 15.66	69.99 ± 9.97	< 0.01
Gender				< 0.01
Male	655 (34.38)	584 (33.31)	71 (46.71)	
Female	1250 (65.62)	1169 (66.69)	81 (53.29)	
Race				< 0.01
Mexican American	206 (10.81)	191 (10.90)	15 (9.87)	
Other Hispanic	149 (7.82)	146 (8.33)	3 (1.97)	
Non-Hispanic White	921 (48.35)	842 (48.03)	79 (51.97)	
Non-Hispanic Black	432 (22.68)	383 (21.85)	49 (32.24)	
Others	197 (10.34)	191 (10.90)	6 (3.95)	
Education				< 0.01
Less than 9th grade	127 (6.67)	109 (6.22)	18 (11.84)	
9-11th grade	137 (7.19)	121 (6.90)	16 (10.53)	
High school graduate/GED or equivalent	423 (22.20)	377 (21.51)	46 (30.26)	
Some college or AA degree	700 (36.75)	655 (37.36)	45 (29.61)	
College graduate or above	518 (27.19)	491 (28.01)	27 (17.76)	
Marital status				< 0.01
Married	1067 (56.01)	985 (56.19)	82 (53.95)	
Widowed	212 (11.13)	173 (9.87)	39 (25.66)	
Divorced	219 (11.50)	205 (11.69)	14 (9.21)	
Separated	74 (3.88)	71 (4.05)	3 (1.97)	
Never married	248 (13.02%)	235 (13.41)	13 (8.55)	
Living with partner	85 (4.46%)	84 (4.79)	1 (0.66)	
PIR	2.80 ± 1.70	2.82 ± 1.70	2.61 ± 1.69	0.11
BMI, kg/m^2^	31.85 ± 8.39	31.85 ± 8.43	31.88 ± 7.99	0.91
Smoke				0.16
Yes	923 (48.45)	841 (47.97)	82 (53.95)	
No	982 (51.55)	912 (52.03)	70 (46.05)	
Alcohol use				0.38
Never	633 (33.23)	589 (33.60)	44 (28.95)	
Moderate	1266 (66.46)	1159 (66.12)	107 (70.39)	
Heavy	6 (0.31)	5 (0.29)	1 (0.66)	
Hypertension				0.67
Yes	1084 (56.90)	995 (56.76)	89 (58.55)	
No	821 (43.10)	758 (43.24)	63 (41.45)	
Diabetes				0.30
Yes	490 (25.72)	455 (25.96)	35 (23.03)	
No	1342 (70.45)	1228 (70.05)	114 (75.00)	
Borderline	73 (3.83)	70 (3.99)	3 (1.97)	
Heart failure				< 0.01
Yes	104 (5.46)	61 (3.48)	43 (28.29)	
No	1801 (94.54)	1692 (96.52)	109 (71.71)	
CHD				< 0.01
Yes	144 (7.56)	119 (6.79)	25 (16.45)	
No	1761 (92.44)	1634 (93.21)	127 (83.55)	
SII	539.58 ± 350.68	540.62 ± 353.50	527.61 ± 317.16	0.89
ALB	41.37 ± 3.49	41.54 ± 3.41	39.41 ± 3.82	< 0.01
ALT	24.50 ± 17.15	24.46 ± 16.08	24.97 ± 26.59	0.20
AST	25.66 ± 15.00	25.48 ± 15.31	27.74 ± 10.60	< 0.01
Cr	81.00 ± 26.43	79.53 ± 24.65	97.96 ± 37.97	< 0.01
Cholesterol	4.82 ± 1.09	4.84 ± 1.08	4.61 ± 1.19	0.10
Triglycerides	1.75 ± 1.22	1.74 ± 1.22	1.88 ± 1.29	0.18
Uric acid	321.97 ± 85.99	320.69 ± 87.26	336.66 ± 68.30	0.03
Serum calcium	2.36 ± 0.10	2.36 ± 0.09	2.37 ± 0.13	0.10
VITD	566.62 ± 466.72	567.23 ± 476.76	559.56 ± 330.57	0.06

Continuous variables are expressed as the mean ± standard deviation, while categorical variables are presented as the number of cases (percentage)

### Association between log VITD and all-cause mortality in asthmatic populations

**Fig. 4:**
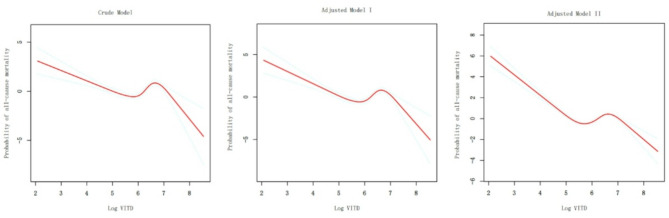
Non-linear relationship of Log VITD and all-cause mortality among participants with asthma (RCS) The crude model was not adjusted for any covariates, provided an initial estimate of the relationship. The adjusted Model I accounted for demographic covariates including age, gender, race, education, marital status, and PIR. Furthermore, Adjusted Model II included additional covariates such as smoking status, alcohol consumption, heart failure, coronary heart disease, diabetes, BMI, SII, ALB, ALT, AST, serum calcium, Cr, cholesterol, triglycerides, and uric acid

Cox regression analysis was employed to investigate the association between vitamin D intake and the risk of all-cause mortality, as presented in Table 4. When Log VITD was treated as a continuous variable, the results from three models indicated that an increase in Log VITD did not significantly reduce the risk of all-cause mortality. Furthermore, when Log VITD was categorized into quartiles, the highest quartile (Q4) compared to the lowest (Q1) also did not significantly lower the risk of all-cause mortality, and the trend test did not yield statistically significant results. This led to the consideration of a potential non-linear relationship between Log VITD and all-cause mortality. The study proceeded to conduct RCS and threshold effect analysis for the three models, as depicted in Fig. 4 and detailed in Table 5, confirming the existence of a non-linear relationship between Log VITD and all-cause mortality.

In the fully adjusted model, the threshold effect analysis identified inflection points at 5.36 (VITD = 213.3 mcg) and 6.52 (VITD = 675.9 mcg). When Log VITD was less than 5.36, an increase in Log VITD was significantly associated with a reduced risk of all-cause mortality with a HR of 0.20 (95% CI: 0.09, 0.45, *p* < 0.01). Within the range of 5.36 to 6.52 for Log VITD, an increase was associated with a significantly elevated risk of all-cause mortality with an HR of 3.32 (95% CI: 1.67, 6.62, *p* < 0.01). Beyond 6.52 for Log VITD, the risk of all-cause mortality was significantly reduced with an HR of 0.24 (95% CI: 0.08, 0.75, *p* < 0.01). The *p*-values for the log likelihood ratio test for both inflection points were less than 0.01, indicating statistical significance.

**Table 4:** HRs (95% CIs) of all-cause mortality according to quartiles of log VITD among participants with asthma

Exposure	Crude Model	Adjusted Model I	Adjusted Model II
	HR (95% CI) *p*	HR (95% CI) *p*	HR (95% CI) *p*
Log VITD (continuous)	1.04 (0.83, 1.29) 0.75	0.95 (0.75, 1.20) 0.67	0.84 (0.65, 1.10) 0.21
Log VITD (quartiles)			
Q1	1	1	1
Q2	1.33 (0.80, 2.22) 0.27	1.20 (0.70, 2.07) 0.50	1.33 (0.72, 2.47) 0.37
Q3	2.11 (1.32, 3.37) < 0.01	1.99 (1.21, 3.25) 0.01	1.64 (0.93, 2.89) 0.09
Q4	1.55 (0.94, 2.55) 0.09	1.45 (0.87, 2.43) 0.16	1.27 (0.70, 2.29) 0.44
*p* for trend	1.18 (1.02, 1.37) 0.02	1.17 (1.00, 1.36) 0.04	1.09 (0.91, 1.29) 0.35

The crude model was not adjusted for any covariates, provided an initial estimate of the relationship. The adjusted Model I accounted for demographic covariates including age, gender, race, education, marital status, and PIR. Furthermore, Adjusted Model II included additional covariates such as smoking status, alcohol consumption, heart failure, coronary heart disease, diabetes, BMI, SII, ALB, ALT, AST, serum calcium, Cr, cholesterol, triglycerides, and uric acid

Q1-Q4 referred to the division of the Log VITD values into quartiles, each representing a 25th percentile range of the distribution

**Table 5:** Threshold effect analysis of log VITD on all-cause mortality among participants with asthma

	Crude Model	Adjusted Model I	Adjusted Model II
	HR (95% CI) *p*	HR (95% CI) *p*	HR (95% CI) *p*
Inflection points	5.26, 6.52	5.28, 6.52	5.36, 6.52
segment 1	0.33 (0.22, 0.50) < 0.01	0.25 (0.15, 0.41) < 0.01	0.20 (0.09, 0.45) < 0.01
segment 2	5.54 (3.00, 10.22) < 0.01	5.47 (2.95, 10.15) < 0.01	3.32 (1.67, 6.62) < 0.01
segment 3	0.22 (0.09, 0.55) < 0.01	0.18 (0.07, 0.46) < 0.01	0.24 (0.08, 0.75) 0.01
*p* for log likelihood ratio test	< 0.01, < 0.01	< 0.01, < 0.01	< 0.01, < 0.01

The crude model was not adjusted for any covariates, provided an initial estimate of the relationship. The adjusted Model I accounted for demographic covariates including age, gender, race, education, marital status, and PIR. Furthermore, Adjusted Model II included additional covariates such as smoking status, alcohol consumption, heart failure, coronary heart disease, diabetes, BMI, SII, ALB, ALT, AST, serum calcium, Cr, cholesterol, triglycerides, and uric acid

Segment 1 represented the curve segment where Log VITD was less than the first inflection point, segment 2 represented the curve segment where Log VITD was between the first and second inflection points, and segment 3 represented the curve segment where Log VITD was greater than the second inflection point

### Association between log VITD and respiratory disease mortality in asthmatic populations

To further investigate the relationship with respiratory disease mortality, which is more closely associated with asthma, the total asthma population of 1,905 individuals was divided into two groups: 32 individuals who died from respiratory diseases and 1,873 individuals who either died from other causes or were censored. A detailed baseline table of this population can be found in Table S2. It shows that among the 1,905 individuals, those who died from respiratory diseases were significantly older (mean age 66.72 years vs. 54.99 years, *p* < 0.01) and had a higher proportion of males (65.62% vs. 33.85%, *p* < 0.01). Additionally, there were significant differences in race, education, marital status, and several other variables between the groups. For example, Non-Hispanic White individuals were overrepresented in the respiratory disease mortality group (93.75% vs. 47.57%, *p* < 0.01), and those who died from respiratory diseases had higher levels of certain blood markers, such as AST (mean 34.97 vs. 25.51, *p* < 0.01) and uric acid (mean 373.81 vs. 321.08, *p* < 0.01). These findings highlight the distinct characteristics of asthma patients who succumbed to respiratory diseases compared to those who did not.

**Fig. 5:**
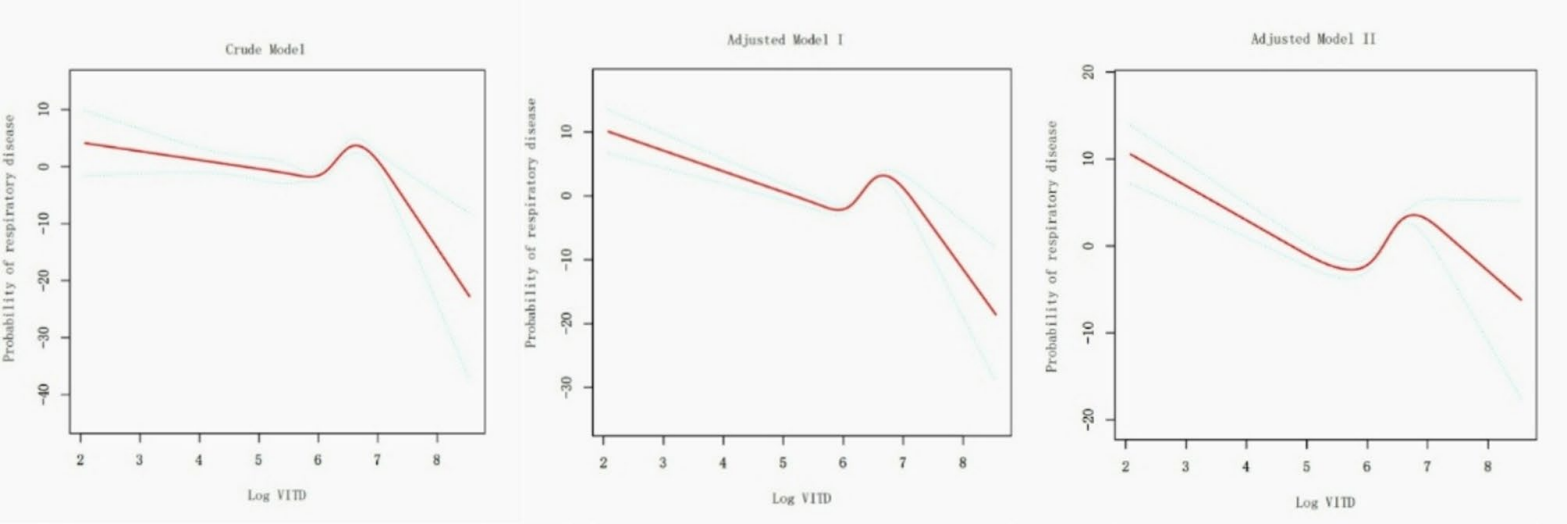
Non-linear relationship of Log VITD and respiratory disease mortality among participants with asthma (RCS) The crude model was not adjusted for any covariates, provided an initial estimate of the relationship. The adjusted Model I accounted for demographic covariates including age, gender, race, education, marital status, and PIR. Furthermore, Adjusted Model II included additional covariates such as smoking status, alcohol consumption, heart failure, coronary heart disease, diabetes, BMI, SII, ALB, ALT, AST, serum calcium, Cr, cholesterol, triglycerides, and uric acid

The non-linear relationship between Log VITD and respiratory disease mortality in the asthmatic population was also studied using RCS (Fig. 5) and threshold effect analysis (Table 6). In the fully adjusted model, an inflection point at 5.48 (VITD = 216.8 mcg) was identified. When Log VITD was below this inflection point, an increase in Log VITD was associated with a decreased probability of respiratory disease mortality, with a hazard ratio of 0.01 (95% CI: 0.00, 0.05, *p* < 0.01). Conversely, when Log VITD exceeded the inflection point, an increase in Log VITD was associated with an increased probability of respiratory disease mortality, with a hazard ratio of 22.38 (95% CI: 8.69, 57.63, *p* < 0.01). The *p*-value for the log likelihood ratio test was less than 0.01, indicating a statistically significant non-linear relationship.

**Table 6:** Threshold effect analysis of log VITD on respiratory disease mortality among participants with asthma

Exposure	Crude Model	Adjusted Model I	Adjusted Model II
	HR (95% CI) *p*	HR (95% CI) *p*	HR (95% CI) *p*
Log VITD (continuous)	2.53 (1.57, 4.08) < 0.01	2.23 (1.09, 4.56) 0.03	3.58 (1.34, 9.55) 0.01
Inflection points	6.52	6.79	5.48
segment 1	567.10 (30.70, 10811.97) < 0.01	28.66 (5.32, 154.53) < 0.01	0.01 (0.00, 0.05) < 0.01
segment 2	0.03 (0.00, 0.36) < 0.01	0.00 (0.00, 0.21) < 0.01	22.38 (8.69, 57.63) < 0.01
*p* for log likelihood ratio test	< 0.01	< 0.01	< 0.01

The crude model was not adjusted for any covariates, provided an initial estimate of the relationship. The adjusted Model I accounted for demographic covariates including age, gender, race, education, marital status, and PIR. Furthermore, Adjusted Model II included additional covariates such as smoking status, alcohol consumption, heart failure, coronary heart disease, diabetes, BMI, SII, ALB, ALT, AST, serum calcium, Cr, cholesterol, triglycerides, and uric acid

Segment 1 represented the curve segment where Log VITD was less than the first inflection point, segment 2 represented the curve segment where Log VITD was between the first and second inflection points, and segment 3 represented the curve segment where Log VITD was greater than the second inflection point

### Sensitivity analyses

**Fig. 6:**
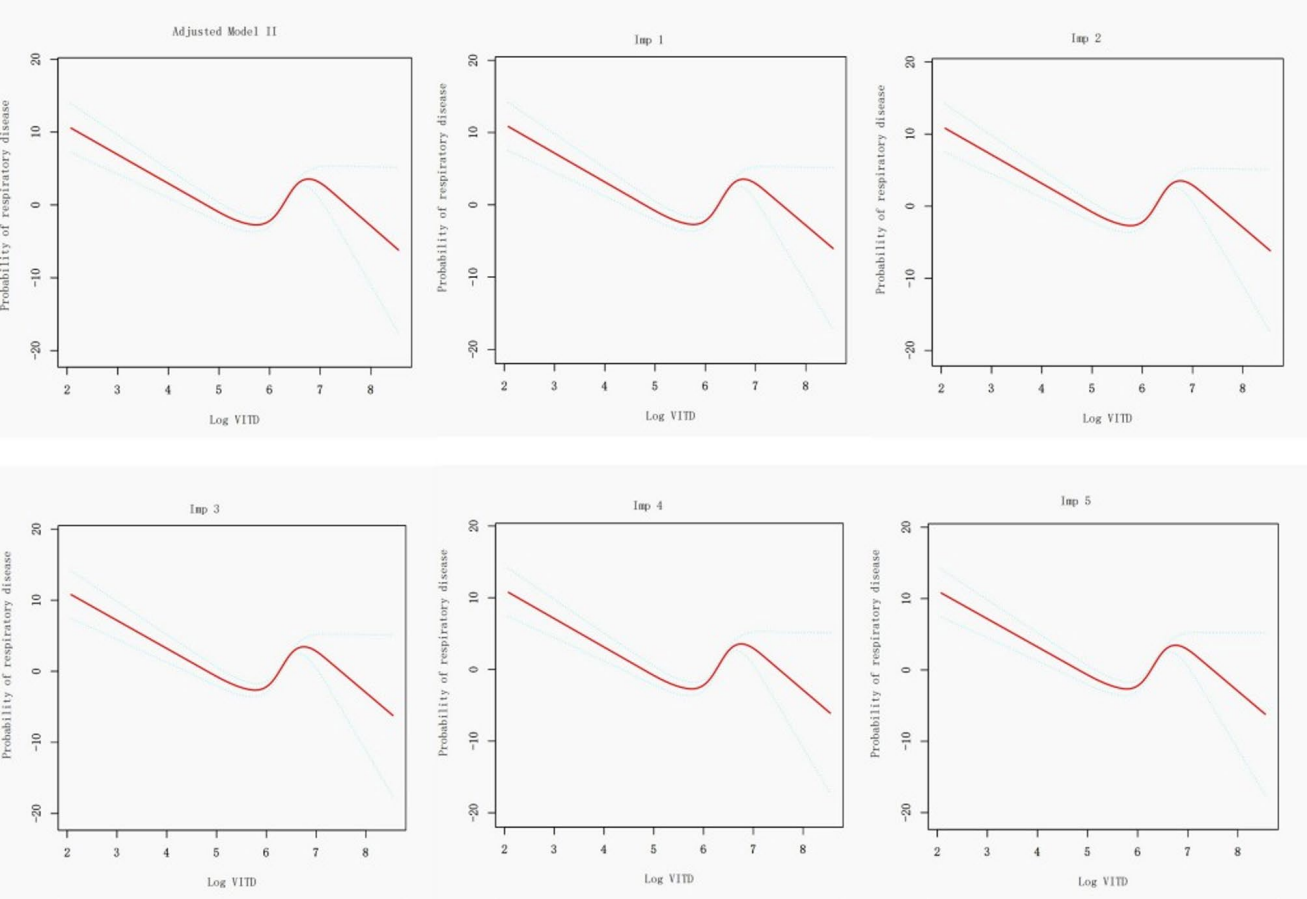
Non-linear relationship of Log VITD and respiratory disease mortality among asthma participants, considering both pre- and pro-imputation data in the adjusted model II (RCS)

To further validate the stability of the relationship between Log VITD and respiratory disease mortality, this study employed multiple imputation for missing data to account for samples with missing covariates. Ultimately, a total of 2,534 asthmatic subjects across five imputed datasets were created, named Imp1 to Imp5, each including 34 individuals with respiratory disease mortality and 2,500 non-deceased individuals. The RCS and threshold effect analysis conducted on these imputed datasets yielded consistent results, confirming the non-linear association and the presence of an inflection point at 5.47 (VITD = 214.6 mcg) or 5.48 (VITD = 216.8 mcg) (as shown in Fig. 6 and Table 7). The relationship between Log VITD and respiratory disease mortality before and after the inflection point was found to be in agreement with the non-imputed dataset, thereby substantiating the stability of the observed relationship.

**Table 7:** Threshold effect analysis of log VITD on respiratory disease mortality in asthma patients considering both pre- and pro-imputation data in the adjusted model II

Exposure	Adjusted Model II	Imp 1	Imp 2	Imp3	Imp4	Imp5
Inflection points	5.48	5.47	5.47	5.48	5.47	5.48
segment 1	0.01 (0.00, 0.05) < 0.01	0.02 (0.00, 0.07) < 0.01	0.02 (0.00, 0.06) < 0.01	0.01 (0.00, 0.06) <0.01	0.01 (0.00, 0.06) <0.01	0.01 (0.00, 0.06) <0.01
segment 2	22.38 (8.69, 57.63) < 0.01	18.57 (7.27, 47.40) < 0.01	18.63 (7.24, 47.92) < 0.01	19.26 (7.44, 49.86) < 0.01	19.17 (7.44, 49.41) < 0.01	19.21 (7.40, 49.83) < 0.01
*p* for log likelihood ratio test	< 0.01	< 0.01	< 0.01	< 0.01	< 0.01	< 0.01

All models adjusted the same covariates, including age, gender, race, education, marital status, PIR, smoking status, alcohol consumption, heart failure, coronary heart disease, diabetes, BMI, SII, ALB, ALT, AST, serum calcium, Cr, cholesterol, triglycerides, and uric acid

Segment 1 represented the curve segment where Log VITD was less than the inflection point, segment 2 represented the curve segment where Log VITD was greater than the inflection point

## Discussion

The findings of this study, consistent with some prior research, indicate a significant inverse association between vitamin D intake and the prevalence of asthma, particularly in males [10, 14]. This association may be attributed to the immunomodulatory and anti-inflammatory effects of vitamin D, which are crucial in the pathogenesis of asthma. Vitamin D, specifically its active form 1,25(OH)2D, has been shown to inhibit the maturation of dendritic cells, reduce the activation of T cells, and promote a shift from a pro-inflammatory Th1/Th17 phenotype to a less inflammatory Th2 phenotype [15, 16]. Additionally, 1,25(OH)2D suppresses the NF-κB pathway, reducing the production of pro-inflammatory cytokines and mitigating oxidative stress [17, 18]. These mechanisms collectively contribute to the observed protective effect of vitamin D against asthma.

The differential impact of sex hormones on the immune system may explain the observed gender-specific association. Estrogen enhances airway hyperresponsiveness and the production of type 2 cytokines through the estrogen receptor-α, while testosterone reduces the proliferation of innate lymphoid cells 2 and the production of type 2 cytokines via the androgen receptor [19]. This suggests that males may benefit more from vitamin D supplementation due to the protective effects of androgens, whereas females may exhibit different responses due to the influence of estrogens. The airways of females exhibit a more complex response to environmental stimuli and inflammation, likely associated with the cyclical variations in sex hormone levels [20].

The relationship between vitamin D intake and mortality in asthmatic populations reveals a complex non-linear association. The inflection points identified in the analysis suggest that maintaining a certain range of vitamin D intake may reduce the occurrence of all-cause or respiratory disease-related deaths. This aligns with previous studies demonstrating that vitamin D deficiency is associated with a higher prevalence of asthma, current wheezing, and reduced lung function, potentially influencing mortality indirectly [21]. Another meta-analysis showed that vitamin D supplementation could reduce the rate of asthma exacerbations requiring systemic corticosteroid treatment [12]. Although this study did not directly assess mortality, it provided evidence of the impact of vitamin D on the severity of asthma, which might be related to reduced mortality. These conclusions are consistent with the findings of this study, suggesting that at low vitamin D intake levels (Log VITD ≤ 5.36–5.47), increasing vitamin D intake can reduce the risk of death in asthmatic populations.

However, discrepancies exist between studies. Nitzan et al.‘s systematic review pointed out that although cross-sectional studies often showed an association between poor vitamin D status and poor asthma control, low 25(OH)D concentrations might not be the cause of poor asthma control [22]. Similarly, Forno et al.‘s study found that vitamin D3 supplementation did not reduce severe exacerbations in children with asthma [11]. Williamson A et al.‘s systematic review concluded that there was no evidence to support that vitamin D supplementation or its hydroxylated metabolites could reduce the risk of asthma exacerbations. Further trials were needed in individuals with frequent severe asthma attacks and those with baseline 25(OH)D concentrations < 25 nmol/L [23]. This study also found that at high vitamin D intake levels (Log VITD > 5.36–5.47), increasing vitamin D intake did not significantly reduce the risk of death in asthmatic populations. This indicated that once vitamin D intake reached a threshold, further increases in vitamin D intake could not reduce mortality in asthmatic populations. The impact of other factors, such as tobacco smoke exposure, air pollution, genetic factors, asthma medication use, and racial differences in patients with adequate vitamin D levels, may require further exploration [24].

The findings suggest that maintaining optimal vitamin D intake levels (Log VITD = 5.36–5.47, VITD = 212.3-214.6 mcg) may reduce mortality risk in asthmatic populations. These results support the development of targeted supplementation strategies for individuals with low vitamin D levels and highlight the importance of public health initiatives to promote adequate vitamin D intake through diet, sun exposure, and supplementation.

### Study strengths and limitations

Study Strengths: The study leveraged the nationally representative NHANES database, which provided a large and diverse cohort for robust statistical analysis and examination of long-term outcomes. The comprehensive data collection and the utilization of advanced statistical techniques, including multiple imputation for missing data, enhanced the reliability and generalizability of the findings.

Study Limitations: The study’s observational nature limits the ability to infer causality. Measurement error in self-reported dietary intakes is also possible. Residual confounding from unmeasured or imperfectly measured variables may exist, and the findings may not be generalizable to populations outside the U.S. context. The definitions of several covariates, such as diabetes and hypertension, relied heavily on self-reports, which may introduce additional bias. Additionally, the lack of serum 25(OH)D measurements limits the precision of evaluating the relationship between vitamin D levels and asthma outcomes. Future studies should consider incorporating such measurements to enhance the robustness of their findings.

## Conclusion

This study leveraged the NHANES database to investigate the relationship between vitamin D intake and the prevalence and mortality of asthma among adults in the United States. Findings indicate that adequate vitamin D intake is associated with a reduced risk of asthma, particularly in males. Additionally, a nonlinear relationship between vitamin D intake and mortality in asthmatic patients was observed, with inflection points identified at Log VITD values of 5.36–5.47 (VITD = 213.3-214.6 mcg). These findings suggest that maintaining vitamin D intake within a specific range may significantly impact the risk of death from asthma.

The study underscores the importance of maintaining appropriate levels of vitamin D in asthma management. The identified inflection points provide a basis for future research to determine the optimal intake levels and their impact on asthma outcomes. These results highlight the need for evidence-based strategies to ensure adequate vitamin D intake in asthma patients, which may contribute to improved clinical outcomes and public health policies.

## Electronic supplementary material

Below is the link to the electronic supplementary material.

Supplementary Material 1

Supplementary Material 2

## Data Availability

No datasets were generated or analysed during the current study.

## Abbreviations

1,25(OH)2D: 1,25-dihydroxyvitamin D
NHANES: National Health and Nutrition Examination Survey
NCHS: National Center for Health Statistics
COPD: Chronic obstructive pulmonary disease
V24h1: Vitamin D intake from the first 24-hour
V24h2: Vitamin D intake from the second 24-hour dietary
VSupp: Total vitamin D intake from dietary supplements over a 30-day period
Mcg: Microgram
VITD: Vitamin D intake
FEV1: Forced expiratory volume in one second
Ml: Milliliters
NDI: National Death Index
PIR: Poverty income ratio
BMI: Body mass index
SII: Systemic inflammatory index
CHD: Coronary heart disease
ALB: Albumin
G/dL: Grams per deciliter
AST: Aspartate aminotransferase
U/L: Units per liter
ALT: Alanine aminotransferase
Mg/dL: Milligrams per deciliter
Cr: Serum creatinine
SD: Standard deviation
Log VITD: Natural logarithm transformation of VITD
GAM: Generalized additive model
RCS: Restricted cubic splines
HR: Hazard ratio
95% CI: 95% Confidence interval
25(OH)D: 25-hydroxycholecalciferol

## References

[1] World Health Organization. Asthma [EB/OL]. Geneva, Switzerland: World Health Organization, 2024 [2024-05-06]. https://www.who.int/zh/news-room/fact-sheets/detail/asthma

[2] Barnett SB, Nurmagambetov TA. Costs of asthma in the united states: direct costs of medical care. J Allergy Clin Immunol. 2011;127(1):13–6. 10.1016/j.jaci.2010.10.020 21211649

[3] CDC. The Economic Burden of Asthma in the United States [EB/OL]. Atlanta, GA: Centers for Disease Control and Prevention, 2024[2024-05-07]. https://www.cdc.gov/asthma/health_costs.htm

[4] Jackson DJ, Sykes A, Mallia P, Johnston SL. Asthma exacerbations: origin, effect, and prevention. J Allergy Clin Immunol. 2011;128(6):1165–74. 22133317 10.1016/j.jaci.2011.10.024 PMC7172902

[5] McEvoy CT, Spindel ER. Pulmonary effects of maternal smoking on the fetus and child: effects on lung development, respiratory morbidities, and life long lung health. Paediatr Respir Rev. 2017;21:27–33. 27639458 10.1016/j.prrv.2016.08.005 PMC5303131

[6] Wright RJ, Subramanian SV, Rich-Edwards JW, et al. Evidence that maternal stress in pregnancy influences childhood asthma risk: results from a prospective birth cohort study. Psychoneuroendocrinology. 2013;38(12):2989–99.

[7] Holick MF. Vitamin D deficiency. N Engl J Med. 2007;357(3):266–81. 17634462 10.1056/NEJMra070553

[8] Liu PT, Stenger S, Li H, et al. Toll-like receptor triggering of a vitamin D-mediated human antimicrobial response. Science. 2006;311(5768):1770–3. 16497887 10.1126/science.1123933

[9] Hewison M. Vitamin D and the immune system. Proc Nutr Soc. 2012;71(1):38–44. 21849106 10.1017/S0029665111001650

[10] Brehm JM, Celedón JC, Soto-Quiros ME, et al. Serum vitamin D levels and markers of severity of childhood asthma in Costa Rica. Am J Respir Crit Care Med. 2009;179(9):765–71. 19179486 10.1164/rccm.200808-1361OC PMC2675563

[11] Forno E, Bacharier LB, Phipatanakul W, et al. Effect of vitamin D3 supplementation on severe asthma exacerbations in children with asthma and low vitamin D levels: the VDKA randomized clinical trial. JAMA. 2020;324:752–60. 10.1001/jama.2020.12384. 32840597 10.1001/jama.2020.12384 PMC7448830

[12] Jolliffe DA, Greenberg L, Hooper RL, et al. Vitamin D supplementation to prevent asthma exacerbations: a systematic review and meta-analysis of individual participant data. Lancet Respir Med. 2017;5(11):881–90. 28986128 10.1016/S2213-2600(17)30306-5 PMC5693329

[13] Katherine F, Retno AS, Qorri A, et al. Vitamin D supplementation decrease asthma exacerbations in children: a systematic review and meta-analysis of randomized controlled trials. Ann Med. 2024;56(1):2400313. 39421966 10.1080/07853890.2024.2400313 PMC11492411

[14] Chinellato I, Piazza M, Sandri M, et al. Vitamin D serum levels and markers of asthma control in Italian children. J Pediatr. 2011;158(3):437–41. 20870246 10.1016/j.jpeds.2010.08.043

[15] Dey SK, Kumar S, Rani D, et al. Implications of vitamin D deficiency in systemic inflammation and cardiovascular health. J Crit Rev Food Sci Nutr. 2024;64(28):10438–55. 10.1080/10408398.2023.2224880 37350746

[16] Cantorna MT, Snyder L, Lin YD, et al. Vitamin D and 1,25(OH)2D regulation of T cells. J Nutrients. 2015;7(4):3011–21. 10.3390/nu7043011 PMC4425186 25912039

[17] Liu W, Zhang L, Xu H, et al. The anti-inflammatory effects of vitamin D in tumorigenesis. Int J Mol Sci. 2018;19(9):2736. 30216977 10.3390/ijms19092736 PMC6164284

[18] Szymczak-Pajor I, Drzewoski J, Śliwińska A. The molecular mechanisms by which vitamin D prevents insulin resistance and associated disorders. 2020;21(18):6644. 10.3390/ijms21186644 PMC7554927 32932777

[19] Ruth PC, Akimichi N, Roma S. Airway androgen receptor expression regulator of sex differences in asthma. J Am J Respir Crit Care Med. 2021;204(3):243–5. 33951407 10.1164/rccm.202104-0869ED PMC8513591

[20] Daniel ERB, Patricia S. Asthma, atopy, and exercise sex differences in exercise-induced. J Exp Biol Med (Maywood). 2021;246(12):1400–9. 10.1177/15353702211003858 PMC8243216 33794694

[21] Zhu YQ, Jing DR, Liang HY, et al. Vitamin D status and asthma, lung function, and hospitalization among British adults. J Front Nutr. 2022 Aug;10:9954768. 10.3389/fnut.2022.954768 PMC9399919 36034921

[22] Nitzan I, Mimouni FB, Nun AB, et al. Vitamin D and asthma: a systematic review of clinical trials. Curr Nutr Rep. 2022;11(2):311–7. 35347665 10.1007/s13668-022-00411-6

[23] Williamson A, Martineau AR, Sheikh A, et al. Vitamin D for the management of asthma. Cochrane Database Syst Rev. 2023;2(2):CD011511. 36744416 10.1002/14651858.CD011511.pub3 PMC9899558

[24] Jessica S, Jennifer P, Augusto AL. Asthma epidemiology and risk factors. J Semin Immunopathol. 2020;42(1):5–15. 10.1007/s00281-020-00785-1 32020334

