# Supplementary material for: The association between vitamin D intake and the prevalence and mortality of asthma in the US adults
Source: Nutr J. 2025 Jul 2;24:103. doi: 10.1186/s12937-025-01171-z (PMC12217372; doi:10.1186/s12937-025-01171-z)
Supplement: Supplementary file 1 — Supplementary Material 1 [file 12937_2025_1171_MOESM1_ESM.docx]

Table S1. ORs, 95% CIs, and p-values for Covariates in the Logistic Regression Models

| Covariates | Adjusted Model II  OR (95% CI) *p* |
| --- | --- |
| Female | 1.19 (1.05, 1.34) 0.01 |
| Year | 0.98 (0.97, 0.98) <0.01 |
| Other Hispanic | 1.17 (0.93, 1.49) 0.19 |
| Non-Hispanic White | 1.09 (0.91, 1,31) 0.34 |
| Non-Hispanic Black | 1.35 (1.10, 1.65) <0.01 |
| Others | 1.14 (0.91, 1.44) 0.25 |
| 9-11th grade | 0.62 (0.47, 0.81) <0.01 |
| High school graduate/GED or equivalent | 0.83 (0.65, 1.05) 0.12 |
| Some college or AA degree | 0.83 (0.66, 1.05) 0.12 |
| College graduate or above | 0.72 (0.56, 0.92) 0.01 |
| Widowed | 1.05 (0.89, 1.26) 0.56 |
| Divorced | 0.89 (0.74, 1.06) 0.15 |
| Separated | 1.28 (0.97, 1.69) 0.08 |
| Never married | 0.97 (0.82, 1.15) 0.70 |
| Living with partner | 0.72 (0.56, 0.93) 0.01 |
| PIR | 0.98 (0.96, 1.03) 0.85 |
| Nonsmoker | 0.89 (0.80, 0.96) 0.01 |
| Moderate drinker | 0.99 (0.90, 1.10) 0.86 |
| Heavy drinker | 0.53 (0.22, 1.24) 0.14 |
| Without heart failure | 0.82 (0.65, 1.03) 0.09 |
| Without diabete | 1.02 (0.91, 1.15) 0.73 |
| Prediabetes | 1.01 (0.77, 1.33) 0.93 |
| Without hypertension | 1.04 (0.94, 1.15) 0.48 |
| BMI | 1.03 (1.03, 1.04) <0.01 |
| ALB | 0.96 (0.95, 0.98) <0.01 |
| Cholesterol | 0.95 (0.90, 0.99) 0.04 |
| Cr | 0.99 (0.99, 1.00) 0.02 |
| Triglycerides | 1.01 (0.97, 1.06) 0.67 |
| SII | 1.00 (0.99, 1.00) 0.59 |
| Uric acid | 0.99 (0.98, 1.00) <0.01 |
| ALT | 0.99 (0.99, 1.01) 0.75 |
| AST | 1.01 (1.00, 1.02) 0.03 |
| Serum calcium | 3.16 (1.85, 5.38) <0.01 |
