# Supplementary material for: The association between vitamin D intake and the prevalence and mortality of asthma in the US adults
Source: Nutr J. 2025 Jul 2;24:103. doi: 10.1186/s12937-025-01171-z (PMC12217372; doi:10.1186/s12937-025-01171-z)
Supplement: Supplementary file 2 — Supplementary Material 2 [file 12937_2025_1171_MOESM2_ESM.docx]

Table S2. Baseline characteristics of asthma patients with death attributed solely to respiratory diseases.

| Characteristics | Overall | Respiratory disease mortality  No Yes | | *p* value |
| --- | --- | --- | --- | --- |
| N（%） | 1905 | 1873 (98.32) | 32 (1.68) |  |
| Age, years | 55.18 ± 15.89 | 54.99 ± 15.92 | 66.72 ± 8.57 | <0.01 |
| Gender |  |  |  | <0.01 |
| Male | 655 (34.38) | 634 (33.85) | 21 (65.62) |  |
| Female | 1250 (65.62) | 1239 (66.15) | 11 (34.38) |  |
| Race |  |  |  | <0.01 |
| Mexican American | 206 (10.81) | 206 (11.00) | 0 (0.00) |  |
| Other Hispanic | 149 (7.82) | 149 (7.96) | 0 (0.00) |  |
| Non-Hispanic White | 921 (48.35) | 891 (47.57) | 30 (93.75) |  |
| Non-Hispanic Black | 432 (22.68) | 431 (23.01) | 1 (3.12) |  |
| Others | 197 (10.34) | 196 (10.46) | 1 (3.12) |  |
| Education |  |  |  | <0.01 |
| Less than 9th grade | 127 (6.67) | 126 (6.73) | 1 (3.12) |  |
| 9-11th grade | 137 (7.19) | 137 (7.31) | 0 (0.00) |  |
| High school graduate/GED or equivalent | 423 (22.20) | 402 (21.46) | 21 (65.62) |  |
| Some college or AA degree | 700 (36.75) | 691 (36.89) | 9 (28.12) |  |
| College graduate or above | 518 (27.19) | 517 (27.60) | 1 (3.12) |  |
| Marital status |  |  |  | <0.01 |
| Married | 1067 (56.01) | 1037 (55.37) | 30 (93.75) |  |
| Widowed | 212 (11.13) | 211 (11.27) | 1 (3.12) |  |
| Divorced | 219 (11.50) | 218 (11.64) | 1 (3.12) |  |
| Separated | 74 (3.88) | 74 (3.95) | 0 (0.00) |  |
| Never married | 248 (13.02) | 248 (13.24) | 0 (0.00) |  |
| Living with partner | 85 (4.46) | 85 (4.54) | 0 (0.00) |  |
| PIR | 2.80 ± 1.70 | 2.82 ± 1.70 | 4.21 ± 0.91 | <0.01 |
| BMI, kg/m^2^ | 31.85 ± 8.39 | 31.85 ± 8.43 | 37.62 ± 9.86 | <0.01 |
| Smoke |  |  |  | 0.05 |
| Yes | 923 (48.45) | 902 (48.16) | 21 (65.62) |  |
| No | 982 (51.55) | 971 (51.84) | 11 (34.38) |  |
| Alcohol use |  |  |  | 0.57 |
| Never | 633 (33.23) | 625 (33.37) | 8 (25.00) |  |
| Moderate | 1266 (66.46) | 1242 (66.31) | 24 (75.00) |  |
| Heavy | 6 (0.31) | 6 (0.32) | 0 (0.00) |  |
| Hypertension |  |  |  | 0.32 |
| Yes | 1084 (56.90) | 1063 (56.75) | 21 (65.62) |  |
| No | 821 (43.10) | 810 (43.25) | 11 (34.38) |  |
| Diabetes |  |  |  | 0.18 |
| Yes | 490 (25.72) | 485 (25.89) | 5 (15.62) |  |
| No | 1342 (70.45) | 1315 (70.21) | 27 (84.38) |  |
| Borderline | 73 (3.83) | 73 (3.90) | 0 (0.00) |  |
| Heart failure |  |  |  | <0.01 |
| Yes | 104 (5.46) | 96 (5.13) | 8 (25.00) |  |
| No | 1801 (94.54) | 1777 (94.87) | 24 (75.00) |  |
| CHD |  |  |  | <0.01 |
| Yes | 144 (7.56) | 135 (7.21) | 9 (28.12) |  |
| No | 1761 (92.44) | 1738 (92.79) | 23 (71.88) |  |
| SII | 539.58 ± 350.68 | 538.23 ± 349.54 | 618.86 ± 410.46 | 0.15 |
| ALB | 41.37 ± 3.49 | 41.42 ± 3.49 | 38.25 ± 2.09 | <0.01 |
| ALT | 24.50 ± 17.15 | 24.51 ± 17.29 | 23.97 ± 2.09 | <0.01 |
| AST | 25.66 ± 15.00 | 25.51 ± 15.06 | 34.97 ± 4.88 | <0.01 |
| Cr | 81.00 ± 26.43 | 81.05 ± 26.62 | 77.99 ± 9.14 | 0.77 |
| Cholesterol | 4.82 ± 1.09 | 4.83 ± 1.10 | 4.46 ± 0.65 | 0.03 |
| Triglycerides | 1.75 ± 1.22 | 1.73 ± 1.20 | 3.20 ± 1.67 | <0.01 |
| Uric acid | 321.97 ± 85.99 | 321.08 ± 86.20 | 373.81 ± 51.44 | <0.01 |
| Serum calcium | 2.36 ± 0.10 | 2.36 ± 0.10 | 2.34 ± 0.09 | 0.94 |
| VITD | 566.62 ± 466.72 | 563.92 ± 469.69 | 724.28 ± 174.86 | <0.01 |

Continuous variables are expressed as the mean ± standard deviation, while categorical variables are presented as the number of cases (percentage).
